# Methicillin-resistant *Staphylococcus aureus* nosocomial infection trends in Hospital Universiti Sains Malaysia during 2002-2007

**DOI:** 10.4103/0256-4947.67077

**Published:** 2010

**Authors:** Hassanain I. Al-Talib, Chan Y. Yean, Karim Al-Jashamy, Habsah Hasan

**Affiliations:** a From the Department of Medical Microbiology and Parasitology, School of Medical Sciences Malaysia, Kubang Kerian, Kelantan, Malaysia; b Faculty of Health and Life Sciences, Management and Science University, Shah Alam, Malaysia

## Abstract

**BACKGROUND AND OBJECTIVES:**

Methicillin-resistant *Staphylococcus aureus* (MRSA) is a major nosocomial pathogen that causes severe morbidity and mortality in many hospitals worldwide. The aim of the present study was to assess the burden of MRSA nosocomial infection, its association with factors of interest, and its antimicrobial susceptibility.

**METHODS:**

This was a retrospective analysis of a database of all *S aureus* that were cultured from patients admitted to the different wards of Hospital Universiti Sains Malaysia (HUSM) over a period of 6 years.

**RESULTS:**

The MRSA infections rate was 10.0 per 1000 hospital admissions. The incidence density rate of MRSA infections during the study period was 1.8 per 1000 patient-days, with annual rates ranging from 0.95 to 3.47 per 1000 patient-days. Duration of hospitalization, previous antibiotic use, and bedside invasive procedures were significantly higher among MRSA than methicillin-sensitive *S aureus* patients (*P*>.05). The highest number of MRSA infections were found in orthopedic wards (25.3%), followed by surgical wards (18.2%) and intensive care units (ICUs) (16.4%). All MRSA isolates were resistant to erythromycin (98.0%), co-trimoxazole (94.0%) and gentamicin (92.0%). Clindamycin was the best antibiotic with only 6% resistance. All MRSA isolates were sensitive to vancomycin.

**CONCLUSION:**

The rate of nosocomial MRSA infection per 1000 admissions was higher than that in other studies. The three factors associated most significantly with acquired MRSA infections included duration of hospitalization, antibiotic use, and bedside invasive procedures. This study confirmed that vancomycin-resistant *S aureus* has not yet been established in HUSM.

*Staphylococcus aureus* is a major pathogen associated with serious community and hospital-acquired diseases. Methicillin-resistant *S aureus*^1^ (MRSA) is responsible for a growing number of nosocomial infections, particularly in critically ill patients.^2^,^3^ MRSA epidemiology seems to be changing, with MRSA strains being implicated in serious infections and nosocomial outbreaks, which appear to be disseminated globally in adult, pediatric, and neonatal intensive care units (ICUs).^4^,^5^ The prevalence of MRSA infection varies from 5% to >50%, depending on the characteristics and size of the hospital. In Australia, 31.9% of the 2908 *S aureus* samples taken from 32 laboratories from all states and territories of the country were resistant to methicillin.^6^ The number of infections with MRSA in United States hospitals rose to nearly 369 000 in 2005.^7^ This increase has been noted in large tertiary-care teaching hospitals and small community hospitals. In Malaysia, a cross-sectional study conducted in Klang Valley in three institutes, Hospital Kuala Lumpur (HKL), Hospital Tengku Ampuan Rahimah, Klang (HTAR) and the Bacteriology Division, Institute for Medical Research (IMR) has revealed that the overall rate of MRSA has increased from 25.7% to 28.7%, 27.9% and 33.0% in 1996, 1998, and 2000, respectively.^8^ Multiple studies carried out in the United States and Europe have shown increased morbidity and mortality from MRSA infection compared with methicillin-susceptible *S aureus* (MSSA) infection in critically ill patients. MRSA has been shown to prolong length of hospital stay, and to result in more adverse outcomes and higher costs.^9^,^10^

These MRSA isolates showed resistance to a wide range of antibiotics, thus limiting the treatment options to very few agents such as vancomycin and teicoplanin.^11^ The mechanism of resistance is an alteration in the target of the antibiotics. MRSA isolates are resistant clinically to all β-lactam antibiotics, and it is important also to note that MRSA is often multidrug resistant and resistant to antibiotics such as macrolides and aminoglycosides, even though the mechanisms of action of these antibiotics are different from those of the β-lactams. Clinical isolates of MRSA that have intermediate resistance to vancomycin, which are called vancomycin-intermediate *S aureus* (VISA), were first identified in patients in Japan in 1996.^12^ Vancomycin has a narrow spectrum of activity that is restricted to most gram-positive bacteria, and it is a drug of choice for the treatment of MRSA infections. In general, fluoroquinolones are active against many gram-positive bacteria, and do not appear to be affected by β-lactamase enzymes or altered penicillin-binding proteins. We undertook the present study to provide epidemiological data, describe infection-associated factors, and establish antimicrobial susceptibility patterns of infection within Hospital Universiti Sains Malaysia (HUSM).

## METHODS

The study was carried out after approval was obtained from the Research and Ethical Committee, School of Medical Sciences, HUSM. HUSM is a 747-bed, referral center for the state of Kelantan and the nearby states, and is the principal teaching hospital of the Universiti Sains Malaysia.

The infection control surveillance system was established in HUSM in 2000, and follows the national infection control surveillance. Diagnosis of nosocomial infection was based on the criteria stated by the Center for Disease Control and Prevention guidelines.^13^ Consequently, HUSM has sufficient infection control personnel to collect the data using standardized protocols, and ample numbers of beds to yield enough cases of healthcare-associated infections for reliable estimation of the incidence and trends over time.^14^

Nosocomial infection means a localized or systemic condition that results from an adverse reaction to an infectious agent or its toxin, which was not present or incubating at the time of hospital admission, which are the definitions adopted by the infection control surveillance system in HUSM.^15^ *S aureus* was identified by standard laboratory procedures. Methicillin resistance was determined according to Clinical and Laboratory Standards Institute (CLSI) guidelines 2005.^16^ Using a cefoxitin disk (30 μg), diffusion screening test, isolates were characterized further by the detection of the penicillin-binding protein 2’ (PBP2’) using the PBP2’ Latex Agglutination Test (MRSA-Screen; Denka Seiken Co, Ltd, Tokyo, Japan). Any *S aureus* isolate that exhibited cefoxitin resistance and PBP2’ positivity was considered to be MRSA. Minimum inhibitory concentration (MIC) against vancomycin was determined to detect any isolate with less susceptibility to vancomycin using the E-test (AB;-Biodisk, Solna, Sweden), according to the manufacturer’s instructions.

Inpatient populations have been followed up for nosocomial and antibiotic-resistant microorganisms as part of routine infection control surveillance. All patients in whom *S aureus* was isolated from 1 January 2002 to 31 December 2007 were analyzed retrospectively. Clinical specimens were processed and analyzed by the clinical microbiology laboratory. Specimens were collected upon suspicion of infection, and screening was performed upon admission to the ICU in patients transferred from other hospitals or health care institutions. Routine MRSA screening for all patients upon admission to the unit was not practiced at the time of the study. Demographic data and factors associated with MRSA infection, such as age, sex, length of stay, previous hospitalization, antibiotic use, and antimicrobial susceptibility, were collected from the Medical Microbiology and Parasitology Laboratory database. No duplicate isolates from the same patient and no environmental strains were included in this study.

Patient characteristics and associated risks were compared for MRSA and MSSA nosocomial infections. The incidence density ratio for MRSA was defined as the total number of new MRSA cases that arose from the defined population in the specified time period, divided by the sum of each individual’s time at risk while remaining free of disease.^17^ Fisher’s exact test was used for categorical data. Multiple logistic regression analysis of variables associated with the acquisition of MRSA nosocomial infection was performed. Covariates that were found to be significant (*P*<.05) in univariate analysis were included. The presence of interactions and colinearity between the covariates of interest was also investigated. Finally, two multiple models were constructed via stepwise selection, using the backward procedure. Performance of the models was assessed via receiver operating characteristic curve analysis to examine discrimination and the Hosmer-Lemeshow test to examine calibration. SPSS version 12.0.1 (SPSS Inc., Chicago, IL, USA) was used for all statistical analyses. Odds ratios (OR) and 95% confidence intervals (95% CI) were calculated. *P*<.05 was considered statistically significant.

## RESULTS

In 196494 admissions to HUSM from 1 January 2002 to 31 December 2007, 1979 patients had MRSA isolated from a clinical specimen during their hospital stay. The MRSA infection rate was 10.0 per 1000 hospital admissions, and annual infection rates ranged from 5.0 to 19.5 per 1000 patient admissions (Table 1). The incidence density rate of MRSA infection during the study period was 1.8 per 1000 patient-days, with annual rates ranging from 0.95 to 3.47 per 1000 patient-days. The highest number of patients with MRSA infection was in 2002, but differences between years were not significant (*P*=.99). Multiple logistic regression was used to analyze the significance of risk factors among MRSA and MSSA patients (Table 2). Duration of hospitalization, previous antibiotic administration, and bedside invasive procedures were significant risk factors for MRSA infection. The mean age of patients with MRSA and MSSA infection was similar (42.3 and 41.7 years, respectively). Excess length of hospital stay was significantly (*P*=.034) greater among MRSA patients (mean [SD], 27 [22.32] days) compared to MSSA patients (13.8 [16.73] days). Patients who had previous antibiotics and those who had undergone bedside invasive procedures were 10 and 20.5 times more likely, respectively, to develop MRSA than MSSA infection. Orthopedic wards had the highest number of MRSA infections (25.3% of the total in HUSM), followed by surgical wards (18.2%), ICUs (16.4%), medical and pediatric wards (11.6%), neurosurgical wards (10.7%), and obstetrics and gynecology wards (6.2%). All MRSA isolates were resistant to penicillin G, followed by erythromycin (98.0%), co-trimoxazole (94.0%), and gentamicin (92.0%) (Table 3). All MRSA isolates were sensitive to vancomycin, with an MIC ≤2 μg/mL, and 85% of MRSA isolates were determined to be multiresistant to different antibiotics.

**Table 1:** MRSA density incidence rate person-days per 1000 admissions according to the year of admission in HUSM.

Year	No. of MRSA	No. of all *S aureus*	No. admission	Average length of stay per year (days)	Density incidence rate MRSA (per 1000 patient days)	Density incidence rate MSSA (per 1000 patient days)	Infection rate for MRSA (per 1000 admissions per year)	*P*
2002	604	2090	31 001	5.6	3.47	8.559	19.5	.99
2003	444	1576	30 671	5.7	2.53	6.475	14.47		
2004	276	1108	31 075	6.1	1.45	4.389	8.88		
2005	219	1079	33 945	5.8	1.11	4.368	6.45	
2006	175	1109	34 396	5.3	0.95	5.123	5.08	
2007	261	1238	35 406	5.3	1.39	5.206	7.37	
									Total	1979	8200	196 494				10.0

**Table 2:** Multiple logistic regressions for the associated risks of MRSA-acquired infections.

Variables	B	Wald	Adjusted ORa	(95%CI)		*P*
				Lower	Upper	
Duration of hospitalization	0.033	13.719	1.034	1.002	0.034	.034
Previous antibiotic	2.313	7.964	10.107	2.027	0.005	.005
Bedside invasive procedures	3.020	4.482	20.493	4.145	<.001	<.001

a Constant -3.456, Adjusted for previous hospitalization and gender

**Table 3:** Sensitivity pattern of 1979 MRSA isolates at the HUSM.

Antibiotics	No.	No. sensitive	No. resistant
Gentamicin	1979	159	1820 (92.0)
Ciprofloxacin	1979	277	1702 (86.0)
Erythromycin	1979	39	1940 (98.0)
Co-trimoxazole	1979	119	1860 (94.0)
Vancomycin	1979	1979	0 (0.00)
Clindamycin	1979	1860	119 (6.0)
Fusidic acid	1979	1365	614 (31.0)
Penicillin	1979	0.0	1979 (100.0)
Rifampicin	1979	1583	396 (20.0)

## DISCUSSION

MRSA and MSSA are responsible for a large proportion of nosocomial infections, and treatment is difficult.^18^ During the past decade, an increasing number of cases of MRSA infection have been encountered globally among healthy community residents.^19^ There also has been a marked increase in the prevalence of MRSA infection reported in Malaysia since the mid-1970s, and this increase has been noted in large tertiary-care teaching hospitals and small community hospitals.^20^ The present study is believed to be the first to determine the MRSA infection rate, incidence density rate, associated factors, and antibiotic susceptibility among inpatients with MRSA infection in HUSM.

Infection rates with MRSA were found to be 10.0 per 1000 hospital admissions, and annual infection rates ranged from 5.0 to 19.5 per 1000 admissions. This figure was a little higher than that reported in a previous retrospective study conducted by the Canadian Nosocomial Infection Surveillance System Program (CNISP) in 2007 in sentinel hospitals, which had a mean 8.62 cases per 1000 admissions.^1^ The present study also showed higher infection rates compared to the monthly rate of MRSA isolates in a previous prospective cohort study in the Royal Prince Alfred Hospital, Australia, which ranged from 4 to 10 per 1000 admissions.^21^ However, our high rate cannot be compared directly with the previous two studies because of the different socio-demographic characteristics of the patients and hospitals.

A review of the literature has revealed a wide range of MRSA infection rates. Among hospitalized patients in the Asia-Pacific region, the rate of MRSA infection was 45.9% and ranged from 5.0% in the Philippines to 79.5% in Hong Kong. This proportion was higher than that reported in Latin America (34.9%), the United States (34.2%), Europe (26.3%), and Canada (5.7%).^2^,^22^ These variations might be ascribed to different populations, interpretation guidelines and culture techniques.

The present study showed a progressive decrease in MRSA infection rates through 2002 to 2006, which might be attributed to the following: new prophylactic measures that have been used by the nosocomial infection committee, such as strict isolation of all patients whether suspected or proven to have MRSA infection; improvements in education, hand hygiene, and the use of hydro-alcoholic solutions; the impact of surveillance; and better quality of care as a whole.

The present study showed that the excess length of hospital stay was significantly longer among MRSA patients (27.0 [22.3] days) compared to MSSA patients (13.8 [16.7] days) (*P*=.034). Therefore, an increase of 1 day in duration of hospitalization will increase the risk of acquisition of MRSA infection by 1.034 times. Our findings were comparable to those in two previous studies. In Belgium, Blot et al reported that the mean length of stay for MRSA patients was 29 days compared to that for MSSA of 10 days.^23^ Another study has found that a length of stay >16 days was associated significantly with MRSA nosocomial infection.^24^ These studies have indicated that those who require a longer duration of hospitalization usually have more severe underlying illness or are critically ill. Therefore, a longer length of hospitalization is considered an associated risk for MRSA infection and carries a greater chance of MRSA transmission among patients.

Previous administration of antibiotics has been shown to be associated significantly with the acquisition of MRSA as compared to MSSA. Oztoprak et al reported that 86% of these patients developed MRSA as compared to 63% of MSSA cases.^25^ A case-control study showed that 41% of MRSA cases received two or more antibiotics compared to 21% of controls.^26^ The overuse and misuse of antibiotics are major contributing factors for bacterial resistance; therefore, antibiotics must be prescribed only when indicated and the drug chosen should have the narrowest spectrum of activity, and be given at an appropriate dose and duration.

The present study detected that bedside invasive procedures were associated significantly with hospital-acquired MRSA infection (*P*<.001). Any patient who has undergone an invasive bedside procedure has a 20.5 times (95% CI: 4.1-101.3) greater risk of acquiring MRSA nosocomial infection, compared to those without such invasive procedures. Some previous studies have supported our results.^27^–^29^

In the univariate analysis, our study showed that both sexes and patients with previous hospitalization within the last year had a significantly higher risk (*P*<.029) for nosocomial acquisition of MRSA, and these results were comparable to the study of Jernigan et al.^30^ Although the mean age of patients with MRSA and MSSA infection was found to be similar, Corea and his co-worker showed that older patients were at an increased risk of MRSA infection.^31^

MRSA was isolated most frequently from orthopedic (25.3%) and surgical (18.2%) wards, which could be attributed to the difficulties in maintaining an adequate standard of hygiene and cross-infection from staff members who may have been asymptomatic carriers of MRSA. Although patients serve as reservoirs for MRSA, medical personnel are often the vectors.^32^ Previous studies have reported that a greater severity of illness upon admission is a predisposing factor for the acquisition of nosocomial MRSA infections.^33^,^34^ This might explain why the ICUs in our study had a high frequency of MRSA (16.4%), which could have resulted from immunosuppression among such patients.

Besides oxacillin, all MRSA isolates encountered in this study were completely resistant to penicillin G, which was similar to a previous study.^35^ Both erythromycin and gentamicin have shown an increase in resistance pattern; an earlier report by Orrett and Land^36^ revealed that MRSA resistance occurred in less than 70% of the MRSA isolates for these two antimicrobial agents in 1997-1998. Probable reasons for this sustained increase are the increase in hospital admissions and poor infection control measures.

It is reassuring that many of the isolates in the present study remained sensitive to the standard anti-MRSA antibiotics available in the hospital, namely rifampicin and fusidic acid. However, other drugs such as co-trimoxazole and ciprofloxacin have been rendered practically useless. With the worldwide use of quinolones, an increasing resistance of *S aureus* to ciprofloxacin has been reported.^37^ Our result confirmed that vancomycin-resistant S aureus has not yet become established in HUSM, unlike in some institutions in Japan,^38^ the United States,^12^ Europe, and the Far East.^39^

We recommend the prudent use of antibiotics, restricting use to only those cases in which it is absolutely necessary, and patients with a high risk of carrying MRSA should undergo active surveillance upon hospital admission. Regular follow-up and proper isolation of all patients suspected or proven to have MRSA infection also should be included in the new prophylactic measures. Finally, a molecular epidemiological study for MRSA genotypes will assist in detection and eventual control of infection.

The retrospective nature of our study did not allow us to collect information on certain factors associated with MRSA infection, such as the type of antibiotics taken during the preceding months or socioeconomic conditions. The molecular evaluation of MRSA clonality could not be performed. The factors determining the choice of initial antibiotic treatment could not be assessed, and nasal *S aureus* colonization was never checked.

The rate of MRSA isolation per 1000 admissions was higher than that in other studies. The three factors associated most significantly with acquisition of MRSA infection were duration of hospitalization, antibiotic use, and bedside invasive procedures. Patients who have these characteristics should be identified and screened vigorously to detect and reduce the occurrence of MRSA in our hospital. This study confirmed that vancomycin-resistant *S aureus* has not yet been established in HUSM.
